# Complete genome sequence of *Nitratireductor aquimarinus* strain MNA2.1 isolated from sediments of the Berre lagoon, France

**DOI:** 10.1128/mra.00259-25

**Published:** 2025-09-10

**Authors:** Nathalie Pradel, Stéphanie Fouteau, Manon Bartoli, Zoé Rouy, Corinne Cruaud, Thierry Nadalig, Pedro H. Oliveira, David Vallenet, Agnès Hirschler-Réa

**Affiliations:** 1 Aix Marseille Univ, Université de Toulon, CNRS, IRD, MIO 27017, Marseille, France; 2 Génomique Métabolique, Genoscope, Institut François Jacob, Commissariat à l’Energie Atomique (CEA), CNRS, Université Evry, Université Paris-Saclay 27048, Gif-sur-Yvette, France; 3 Genoscope, Institut François Jacob, CEA, Université Paris-Saclay 27048, Evry, France; 4 Génétique Moléculaire, Génomique, Microbiologie (GMGM), Université de Strasbourg, UMR 7156 CNRS 27083, Strasbourg, France; California State University San Marcos, San Marcos, California, USA

**Keywords:** *Nitratireductor aquimarinus*, salt stress, Berre lagoon, genome

## Abstract

We report the complete genome sequence of *Nitratireductor aquimarinus* strain MNA2.1, isolated from coastal sediments of the Berre lagoon, France. The genome consists of a 3,866,286 bp circular chromosome and a megaplasmid of 715,144 bp. The genome contains the *nar* genes for incomplete nitrate reduction and genes encoding systems for salinity stress tolerance.

## ANNOUNCEMENT

The Berre lagoon is a shallow ecosystem gathering steel industries and oil refineries, connected to the Mediterranean Sea, in southeastern France. It receives freshwater from three rivers, inducing high salinity level variations along the year (1). In addition, these rivers carry an annual mean load of more than 600 tons of total N in the lagoon. The genome of *Nitratireductor sp*. 1Nfss2.1, isolated from this lagoon, provides light on the adaptation of this genus to this ecosystem. Edge sediments were collected with a piston of 5 cm diameter at 20 cm depth (43°29′31.7″N 5°12′21.5″E). Growth was performed at 30°C in a serum bottle from 5 mL of sediments in 50 mL of medium containing 0.1 g.L^−1^ KH_2_PO_4_, 0.1 g.L^−1^ K_2_HPO_4_, 0.2 g.L^−1^ NH_4_Cl, 7 g.L^−1^ NaCl, 1.3 g.L^−1^ MgCl_2_.6H_2_O, 0.1 g.L^−1^ KCl, 0.05 g.L^−1^ CaCl_2_.2H_2_O, 2 g.L^−1^ yeast extract, 0.1% trace element solution SL-10 (DSMZ 320), 0.1% Se-Ni-Mo-W solution (DSMZ 385), 1% Balch’s vitamins (2), and 20 mM KNO_3_, under N_2_ atm. The pH was 7.5. After 15 days of incubation, three subcultures were performed every 5 days in the same conditions (1/10 dilution factor). Then, for strain isolation, 10^−2^ serial dilutions were performed from the third subculture, in 5 mL of solid medium (liquid medium containing 1.5% agar), in Hungate tubes, using the roll-tubes technique (3). Briefly, 5 mL of agar medium, inoculated with 0.05 mL of the third subculture, were distributed in serial dilutions from 10^−2^ to 10^−8^ as a thin layer, with a tube spinner over the internal surface of Hungate tubes charged with an N_2_ atmosphere. Colonies developed after 7 days in the 10^−6^ dilution tube. A single colony was then grown in 2 mL of liquid medium in Hungate tube, and then from this culture, 10^−2^ serial dilutions up to extinction (dilution 10^−8^) were prepared in 5 mL of liquid medium in Hungate tubes (1/10 dilution factor). Serial dilutions in the same liquid conditions were repeated three times, every 48 h, from the 10^−6^ dilution tube. DNA was extracted from 5 mL of the subculture (1/10 dilution factor) of this last axenic 10^−6^ dilution culture, using the Wizard Genomic DNA Purification Kit, according to the manufacturer’s recommendations (Promega).

Genome sequencing was carried out using both Illumina and Nanopore Technologies. For Illumina, 250 ng of DNA was sonicated to a mean size of 380 bp using a Covaris E210 Focused-ultrasonicator, and profiles were visualized on an Agilent Bioanalyzer DNA chip. The fragments were end-repaired and 3′-adenylated (NEBNext DNA Sample Prep Master Mix, New England Biolabs) and ligated to NEXTflex barcodes (Bioo Scientific Corporation) (4). The ligated product was amplified by 12 PCR cycles using KAPA HiFi Library Amplification (KAPABIOSYSTEMS) and purified with 0.6 × AMPureXP (Beckman Coulter). The library was sequenced on an Illumina NovaSeq 6000 with a NovaSeq Reagent Kit-v2 (2 × 150 bp). A total of 3,375,530 paired-end reads were obtained. Low-quality nucleotides (Q < 20), adaptors, and sequences < 30 nt were removed using fastx_toolkit-v0.0.14 (http://hannonlab.cshl.edu/fastx_toolkit).

Nanopore sequencing was performed according to Oxford Nanopore Technologies (Oxford, UK). Library was prepared with 400 ng DNA, following the Native Barcoding Kit 24-v14 protocol with the SQK-NBD114.24 ligation kit. The library was sequenced without any size selection using an R10.4.1 flow cell and GridION device with MinKNOW-v6.0.14 and Guppy-v7.4.14 + d47971 cbb software. A total of 118,620 reads were obtained with an N50 of 15 kb. Assembly was performed with Flye-v2.9.5 (5) on long reads filtered with Filtlong-v0.2.1 (min length 6,000, keep_percent 90; https://github.com/rrwick/Filtlong), resulting in an assembly in two loop-edges representing a circular chromosome and a circular megaplasmid. Polishing was done with long reads using Medaka-v2.0.1 (https://github.com/nanoporetech/medaka) and with Pypolca-v0.3.1 (6). Default settings were used for all software. Annotation was performed at the Microscope platform-v3.17.3 (7).

The genome comprised a 3,866,286 bp circular chromosome and a megaplasmid of 715,144 bp. The average G + C content of the chromosome was 62.21%, and 4,375 coding DNA sequences (CDSs) were predicted. The average G + C content of the megaplasmid was 61.78% with 664 predicted CDSs. NCBI BLASTn analysis indicated 99.9% identity over 96% length of the 16S rRNA sequence of *N. aquimarinus* CL-SC21^T^ isolated from a diatom culture from the east coast of Korea (8). The average nucleotide identity value with the closest sequenced genome of*N. aquimarinus* KCTC-32151 was 98.3% (EZ BioCloud, 21 April 2025). The genome of MNA2.1 contains the *nar* genes encoding for respiratory nitrate reduction to nitrite, and genes for ectoine and betaine biosynthesis, and betaine and choline uptake systems, involved in salinity stress tolerance. It may be advantageous to counter the effects of change in salinity in the Berre lagoon.

## Data Availability

The whole genome project and the raw data have been deposited in the European Nucleotide Archive under the numbers PRJEB86991 and ERA32312406, respectively. The chromosome and the megaplasmid sequences are available at ENA under the accession no. OZ244555-OZ244556.
